# International Brazilian Journal of Urology highlights experimental studies in Infertility in this number

**DOI:** 10.1590/S1677-5538.IBJU.2024.04.01

**Published:** 2024-07-12

**Authors:** Luciano A. Favorito

**Affiliations:** 1 Unidade de Pesquisa Urogenital Universidade do Estado do Rio de Janeiro Rio de Janeiro RJ Brasil Unidade de Pesquisa Urogenital - Universidade do Estado do Rio de Janeiro - Uerj, Rio de Janeiro, RJ, Brasil;; 2 Serviço de Urologia Hospital Federal da Lagoa Rio de Janeiro RJ Brasil Serviço de Urologia, Hospital Federal da Lagoa, Rio de Janeiro, RJ, Brasil

The July-August number of Int Braz J Urol presents original contributions with a lot of interesting papers in different fields: Robotic Surgery, Prostate Cancer, Infertility, Endourology, Basic Research, Renal Transplantation, Erectile dysfunction, Testicular cancer and Vesico-vaginal fistula. The papers came from many different countries such as Brazil, Italy, Kazakistan, India, Singapure and USA, and as usual the editor ´s comment highlights some of them. The editor in chief would like to highlight the following works:

Dr. Iskakov and collegues from Kasakstan, presented in page 386 (1) a nice review about the treatment of erectile dysfunction by intracavernosal administration of mesenchymal stem cells in patients with diabetes mellitus and concluded that standardising the preparation of stem cells and the treatment method using a large sample size is essential to introduce such a method as an extremely promising therapy for this delicate issue in men into practical medicine. The practical value of the study lies in the systematisation of information on different sources of mesenchymal stem cells for treating erectile dysfunction.

Dr. Thakker and Collegues from USA performed in page 398 (2) an interesting narrative review about the Current State of Robot-assisted Laparoscopic Salvage prostatectomy (sRARP) and concluded that Advances in the robotic platform and improvement in robotic experience have resulted in acceptable complication rates after sRARP. However, oncologic and functional out- comes after sRARP in both the post-radiation and post-ablation settings are worse compared to pRARP. Thus, when engaging in shared decision making with patients regarding the initial management of localized prostate cancer, patients should be educated regarding oncologic and functional outcomes and complications in the case of biochemically recurrent prostate cancer that may require sRARP.

In page 415 (3) we can obseve an important sytematic review performed by Dr. Melão and collegues about the Primary Retroperitoneal Lymph Node Dissection (RPLNDs) for Clinical Stage II A/B Seminomas and concluded that primary RPLNDs for treating stage IIA/B seminomas have favorable RFS rates, with low complication and recurrence rates. These findings provide evidence that this surgery is a vi- able alternative therapy for these patients.

Dr. Carvalho and collegues from Brazil permorfed in page 433 (4) a nice experimental study about the moment of induction and duration of experimental varicocele in rats: effects on semen quality and concluded that in this study conducted on rats, we conclude that varicocele adversely affects sperm, particularly its function. However, we did not observe a negative progressive effect on sperm.

Dr. Porcaro and collegues form Italy performed in page 450 (5) a nice study about prognostic impact of the 2012 Briganti nomogram on prostate cancer (PCa) progression in intermediate-risk (IR) patients presenting with PSA <10ng/mL, ISUP grade group 3, and clinical stage up to cT2b treated with robot assisted radical prostatectomy eventually associated with extended pelvic lymph node dissection and concluded that in IR PCa patients with PSA <10ng/mL, ISUP grade group 3, and clinical stage up to cT2b, the 2012 Briganti nomogram independently predicts PCa progression. In this challenging subset of patients, this tool can identify prognostic subgroups, independently by upgrading issues.

Dr. Giulioni and collegues from Italy performed in page 459 (6) a interesting study about the incidence of the most common intra- and early postoperative complications following RIRS in a large series of patients with kidney stones and concluded that RIRS has a good safety profile but bleeding requiring transfusions, ureteric injury, fever, and sepsis are still the most common complications despite advancements in technology.

Dr. Leite and collegues from Brazil performed in page 470 (7) a nice study about the clinical outcomes prediction in kidney transplantation by use of biomarkers from hypotermic machine perfusion and concluded that the concentrations of neutrophil gelatinase- associated lipocalin (NGAL) and LDH during machine perfusion could assist transplant physicians in improving the allocation of donated organs and making challenging decisions regarding organ discarding. Further, larger-scale studies are required.

Dr. Pereira and collegues from Brazil performed in page 480 (8) a experimental study about the morphological and stereological parameters of the testicles in mice exposed to bisphenol S and/or high-fat diet-induced obesity and concluded thatanimals exposed to bisphenol S and/or high-fat diet-induced obesity presented important structural alterations in testicular morphology.

Dr. Faria and collegues from Brazil performed in page 489 (9) the cover of this edition a original study about mesentery-Sparing Technique: a New Intracorporeal approach for Urinary Diversion in Robot-Assisted radical cystectomy and concluded that the mesentery-sparing technique outlined herein offers an alternative method for preserving the vascularization of intestinal segments and reducing the risk of intestinal complications in ntracorporeal urinary diversion during Robotic-assisted radical cystectomy .

The Editor-in-chief expects everyone to enjoy reading.
